# Intrinsically-disordered N-termini in human parechovirus 1 capsid proteins bind encapsidated RNA

**DOI:** 10.1038/s41598-018-23552-7

**Published:** 2018-04-11

**Authors:** Shabih Shakeel, James D. Evans, Mark Hazelbaker, C. Cheng Kao, Robert C. Vaughan, Sarah J. Butcher

**Affiliations:** 1 Institute of Biotechnology and Research Programme in Molecular and Integrative Biosciences, Faculty of Biological and Environmental Sciences, University of Helsinki, 00790 Helsinki, Finland; 2 Department of Molecular and Cellular Biochemistry, Indiana University, Bloomington, IN 47405 USA; 3 Present Address: MRC Laboratory of Molecular Biology, Francis Crick Ave, Cambridge, CB2 OQH UK

## Abstract

Human parechoviruses (HPeV) are picornaviruses with a highly-ordered RNA genome contained within icosahedrally-symmetric capsids. Ordered RNA structures have recently been shown to interact with capsid proteins VP1 and VP3 and facilitate virus assembly in HPeV1. Using an assay that combines reversible cross-linking, RNA affinity purification and peptide mass fingerprinting (RCAP), we mapped the RNA-interacting regions of the capsid proteins from the whole HPeV1 virion in solution. The intrinsically-disordered N-termini of capsid proteins VP1 and VP3, and unexpectedly, VP0, were identified to interact with RNA. Comparing these results to those obtained using recombinantly-expressed VP0 and VP1 confirmed the virion binding regions, and revealed unique RNA binding regions in the isolated VP0 not previously observed in the crystal structure of HPeV1. We used RNA fluorescence anisotropy to confirm the RNA-binding competency of each of the capsid proteins’ N-termini. These findings suggests that dynamic interactions between the viral RNA and the capsid proteins modulate virus assembly, and suggest a novel role for VP0.

## Introduction

Human parechoviruses (HPeV) are important human pathogens for which we lack antivirals or vaccines. They have a positive-sense, single-stranded RNA genome and belong to the *Picornaviridae* family. The mature virion is icosahedrally-symmetric with a triangulation number of T = 1 (pseudo T = 3) and is composed of capsid proteins VP0, VP1 and VP3^1–4^. Unlike in other picornaviruses, the parechovirus VP0 is not proteolytically cleaved in the final maturation of the virions^5^. There is also an extensive network of VP0 N-termini on the inner capsid surface that enhance inter-pentamer stability, along with an annulus of VP3 termini under the vertex^3^.

Regions of structured RNA were recently identified as packaging signals (PSs) that interact with VP1 and VP3 in the HPeV virion^4^. Upon interaction with viral pentameric assembly intermediates, these PSs drive capsid assembly. Multiple VP1 and VP3 residues were found to contact the viral RNA in the atomic models of HPeV1 (PDB: 4Z92 & 5MJV)^3,4^. When these residues were mutated to alanine, virus assembly was prevented^4^. The atomic models do not cover the complete sequences of the capsid proteins or the full genome. The N-terminal regions of all three capsid proteins were apparently disordered^3,4^. The virion population may contain multiple states of both the RNA and the capsid, as was recently observed for bacteriophage MS2^6–8^. Hence, we expect that there are more RNA-protein interactions to be discovered in the virion. More direct methods could be utilized to identify the regions of the HPeV1 capsid that interact with the encapsidated RNA. One such method is reversible cross-linking, affinity purification, and peptide-mass fingerprinting (RCAP) which has previously been used to map protein-nucleic acid interaction sites. RCAP has been successfully used to map regions of the capsid protein that interact with the virion RNA in brome mosaic virus, adenovirus, and bacteriophage MS2^9–12^. The MS2 protein-RNA interactions identified by the RCAP assay have since been confirmed in asymmetric cryoEM reconstructions of MS2^6,13^.

Here we mapped regions within the HPeV1 capsid proteins that interact with the encapsidated RNA using RCAP. Several regions within VP1 and VP3 were found to interact with the RNA. Surprisingly, VP0 was also identified to contact the genomic RNA within the HPeV virion. The N-terminal regions of all capsid proteins not visualized in the HPeV1 atomic model apparently contact viral RNA. Recombinantly-expressed VP0 and VP1 protein were shown to bind both the full genome as well as several sub-genome length RNAs. Curiously, the RNA binding profile of VP0 differed dramatically when analyzed in the context of the virion or with the recombinant VP0. RNA fluorescence anisotropy was used to confirm that the disordered capsid N-termini indeed contact the genome. We propose that these highly disordered regions are important for both RNA binding and the co-assembly of the virion genome and capsid proteins.

## Results and Discussion

**Figure 1:**
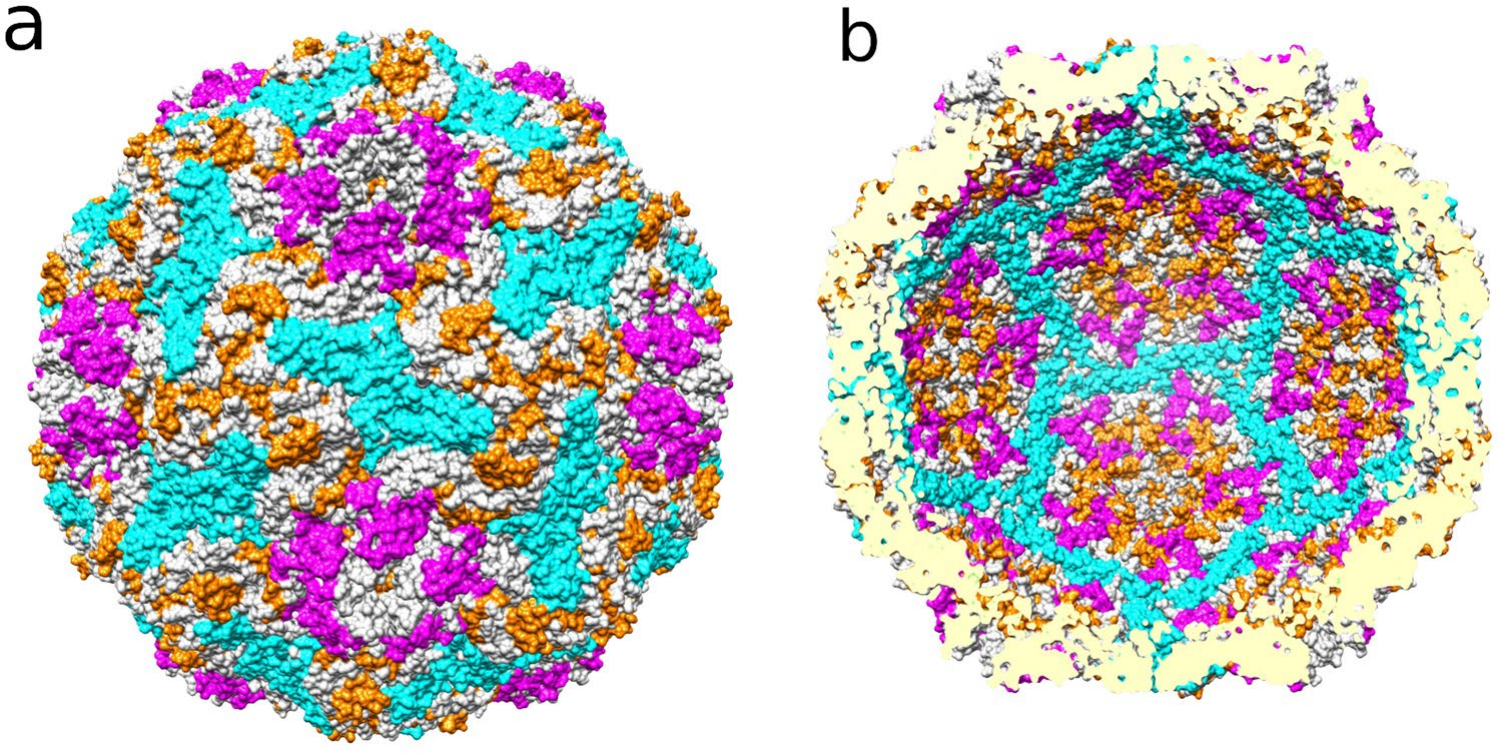
Results of whole-virion HPeV1 RCAP. The top hits arising from each of the capsid proteins from the virion RCAP are shown on the HPeV1 model (PDB: 4Z92). (**a**) Visualized on the outer surface of the capsid, all but one hit span the capsid and are exposed on the inner surface too. (**b**) Regions identified by RCAP visualized on the inner capsid surface. The peptides from VP0 are cyan, VP1 are magenta and VP3 are orange. Note that the N-termini of VP0 and VP1, although in the top hits, are disordered and therefore not seen in this representation.

RCAP analysis on whole HPeV1 virions identified that several sequences from VP0, VP1 and VP3 contacted the encapsidated RNA (Table 1; Fig. 1; Supplementary Figure 1). The RCAP assay used trypsin, which preferentially cleaves C-terminal to lysines and arginines^11,14^. Within these peptides, several lysines and arginines were not cleaved, likely due to their crosslinking to RNA^15^. These missed cleavages increase our confidence in the assignment of the protein sequences that contact the RNA. These RCAP peptides were mapped primarily to the inner surface of the capsid from the HPeV1 atomic model, or to disordered regions likely to be in the inner cavity of the capsid. Of the peptides that appear on the outer surface of the capsid, they all actually spanned the capsid and thus were exposed to the encapsidated RNA, with the exception of one short peptide (VP1 91–98; Supplementary Table 1; Supplementary Movie 1). This peptide may have more than one conformation in the capsids present in the preparation, there could have been some partial virion dissociation or RNA extrusion from the virion. All of the residues predicted to bind RNA in one or both of the atomic models^3,4^ were present in, or within 3 residues, to peptides identified by RCAP. Residues 17, 18, 41, 44, 55–58 and 68 in VP3 and 202 in VP1 were identified to contact RNA, both using RCAP, and X-ray crystallography of the HPeV1 virion (PDB: 4Z92)^3,4^. Residues 19 and 21 in VP3 and 203 and 205 in VP1 were found to be in RNA-interacting regions in the atomic models, but were not directly identified with high confidence by RCAP. Rather, these residues were found in close proximity to residues which were identified. In light of this confirmation of previous data, we now went on to consider the other RCAP results for which there was no *a priori* data. Strikingly, several regions which are disordered in the HPeV1 atomic model were identified by RCAP to contact RNA, specifically: residues 6–31 of VP0, 1–23 of VP1; and 11–14 for VP3 (Table 1).

**Table 1:** Summary of regions from the HPeV1 virions that crosslinked to the encapsidated genomic RNA.

Capsid protein	Amino acid sequence^a^
VP0	METIK**SIADM ATGVVSSVDS TINAVNEKVE SVGNEIGGNL LTKVADDASN ILGPNCFATT AEPENK**NVVQ ATTTVNTTNL TQHPSAPTMP FSPDFSNVDN FHSM**AYDITT GDkNPSkLVR LETHEWTPSW ARGYQITHVE LPKVFWDHQD** **kPAYGQSRYF AAVR**CGFHFQ VQVNVNQGTA GSALVVYEPK PVVTYDSKLE FGAFTNLPHV LMNLAETTQA DLCIPYVADT NYVK**TDSSDL GQLK**VYVWTP LSIPTGSANQ VDVTILGSLL QLDFQNPR**VF AQDVNIYD*N***
VP1	**NSWGSQMDLT DPLCIEDDTE NCK**QTMSPNE LGLTSAQDD**G PLGQEkPNYF LNFRSMNVDI FTVSHTkVDN LFGRAWFFME HTFTNEGQWR VPLEFPK**QGH GSLSLLFAYF TGELNIHVLF LSERGFLR**VA HTYDTSNDR**V NFLSSNGVIT VPAGEQMTL**S APYYSNkPLR** TVRDNNSLGY LMCKPFLTGT STGK**IEVYLS** **L*R****C*P*N*FFFPL PAPKVTSSRA LRGDMANLTN QSPY
VP3	APNGKKKNWK **kIMTMS*TK****Y*K *W*TRTK**IDIAE GPGSMNMANV** ***L*CT*T*GAQSVA LVGE*RAFY*DP R**TAGSK**S*R*FD DLVKIAQLFS VMADSTTPSE NHGVDAK**GYF KWSATTAPQS IVHRNIVYLR LFPNLNVFVN SYSYFRGSLV LR**LSVYASTF NR**GRLRMGFF PNATTDSTST LDNAIYTICD IGSDNSFEIT IPYSFSTWMR **kTNGHPIGLF QIEVLNRLTY NSSSPSEVYC IVQGK**MGQDA R**FFCPTGSVV TFQ**

^a^The residues from the twelve most intense peaks identified are in bold. The missed cleavages are shown in lower case letters and highlighted in bold-underline. The disordered regions not identified in the atomic model are underlined. Residues hypothesized to contact viral RNA in the atomic model (PDB ID:4Z92) are highlighted in italic.

To confirm the RNA-contacting residues in VP1, we expressed and purified recombinant VP1 from *E. coli* and confirmed that the protein was folded using Fourier transformed infrared (FTIR) spectroscopy (Supplementary Figure 2). The obtained spectra showed amide I peaks at 1620 cm^−1^, characteristic of β-sheets^16^. The spectrum of VP1 showed an additional amide I peak at 1656 cm^−1^ characteristic of α-helices arising from the thioredoxin tag. Amide II peaks were seen in the VP1 spectra at 1530 cm^−1^. To obtain a reference spectrum of denatured peptide, aliquots of VP0 or VP1 fractions were mixed 1:1 with 100% trichloroacetic acid (TCA) and assayed as wet drops to obtain spectra of denatured protein. TCA treatment caused the two amide I peaks to collapse into a single broad peak at 1654 cm^−1^ characteristic of disordered peptide, and the complete ablation of the amide II peak (Supplementary Figure 2).

**Figure 2:**
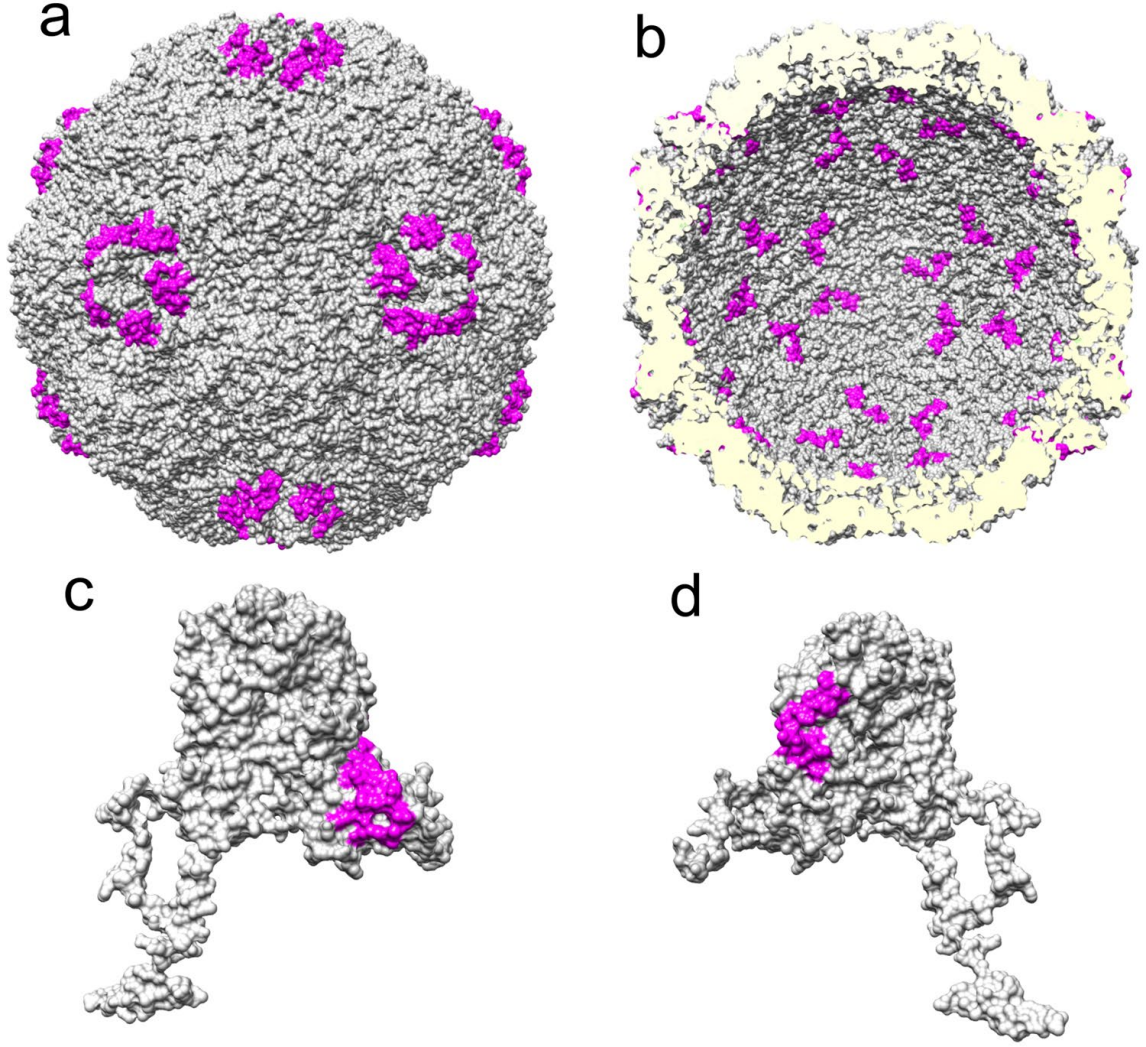
RCAP of capsid protein VP1. RCAP results of recombinantly-expressed VP1 are shown on the HPeV1 model (PDB: 4Z92). (**a**) Peptides from VP1 identified by RCAP that are localized to the outer capsid surface (**b**) Peptides from VP1 identified by RCAP that localize to the inner capsid surface. (**c**) Locations of the peptides that contact RNA on a monomer of VP1 with a view corresponding to the outer capsid surface whereas (**d**) A view of VP1 that corresponds to the inner capsid surface.

Purified VP1 was then mixed with RNA from the sub-genomic HPeV1 regions and subjected to RCAP analysis. All the RNA-binding peptides identified were subsets of the peptides identified in VP1 from RCAP of the virion (Table 2; Fig. 2). Compared to RCAP using whole virions, fewer peptides were identified using the recombinant VP1 protein. The coverage dropped from 47% to 15%, and the observed missed cleavages dropped from 3 to 1. Peptides containing residues 55–74 and 55–90, identified in the atomic model as being on the inner capsid surface and in whole virion RCAP to bind viral RNA, were recovered in this experiment and carried the missed cleavage at residue 67 originally observed in the whole virion RCAP analysis. However, the N-terminal disordered region and putative RNA-binding residue 202 were both notably missing. Different sub-genomic RNAs were employed to determine if protein-RNA contacts were dependent on transcript length, and this turned out to not be the case. We could not verify the RNA binding of recombinant VP3 on its own, as it could not be purified in a folded state.

**Table 2:** Summary of peptides derived from capsid protein VP1 that were crosslinked to sub-genomic HPeV1 RNA.

Sub-genomic sequence length (n.t.)	Amino acid sequence^a^
1576	NSWGSQMDLT DPLCIEDDTE NCKQTMSPNE LGLTSAQDDG PLGQEKPNYF LNFR**SMNVDI FTVSHTkVDN LFGR**AWFFME HTFTNEGQWR VPLEFPKQGH GSLSLLFAYF TGELNIHVLF LSERGFLRVA HTYDTSNDRV NFLSSNGVIT VPAGEQMTLS APYYSNKPLR TVRDNNSLGY LMCKPFLTGT STGKIEVYLS *LRC*P*N*FFFPL PAPKVTSSRA LRGDMANLTN QSPY
2335	NSWGSQMDLT DPLCIEDDTE NCKQTMSPNE LGLTSAQDDG PLGQEKPNYF LNFR**SMNVDI FTVSHTkVDN** LFGRAWFFME HTFTNEGQWR VPLEFPKQGH GSLSLLFAYF TGELNIHVLF LSERGFLRVA HTYDTSNDRV NFLSSNGVIT VPAGEQMTLS APYYSNKPLR TVRDNNSLGY LMCKPFLTGT STGKIEVYLS *LRC*P*N*FFFPL PAPKVTSSRA LRGDMANLTN QSPY
3037	NSWGSQMDLT DPLCIEDDTE NCKQTMSPNE LGLTSAQDDG PLGQEKPNYF LNFR**SMNVDI FTVSHTkVDN LFGRAWFFME HTFTNEGQWR** VPLEFPKQGH GSLSLLFAYF TGELNIHVLF LSERGFLRVA HTYDTSNDRV NFLSSNGVIT VPAGEQMTLS APYYSNKPLR TVRDNNSLGY LMCKPFLTGT STGKIEVYLS *LRC*P*N*FFFPL PAPKVTSSRA LRGDMANLTN QSPY

^a^The RCAP peptides identified are in bold. The missed cleavages are shown in lowercase and highlighted in bold-underline. The disordered regions not identified in the atomic model are underlined. Residues hypothesized to contact viral RNA in the atomic model highlighted in italic.

Unexpectedly, VP0 was found to contact the encapsidated RNA in virions. To further confirm this observation, we expressed and purified recombinant VP0 and confirmed that the protein was folded using FTIR spectroscopy as for VP1 (Supplementary Figure 2). Amide I absorption was observed at 1626 cm^−1^, indicating the presence of β sheets in the protein^15^ and amide II at 1517 cm^−1^. No absorption for α helices was observed due to the lack of the thioredoxin tag. Chemically denaturing the purified VP0 resulted in a shift in the amide I absorption to higher wavenumbers and the loss of signal at amide II, as was the case for VP1.

**Figure 3:**
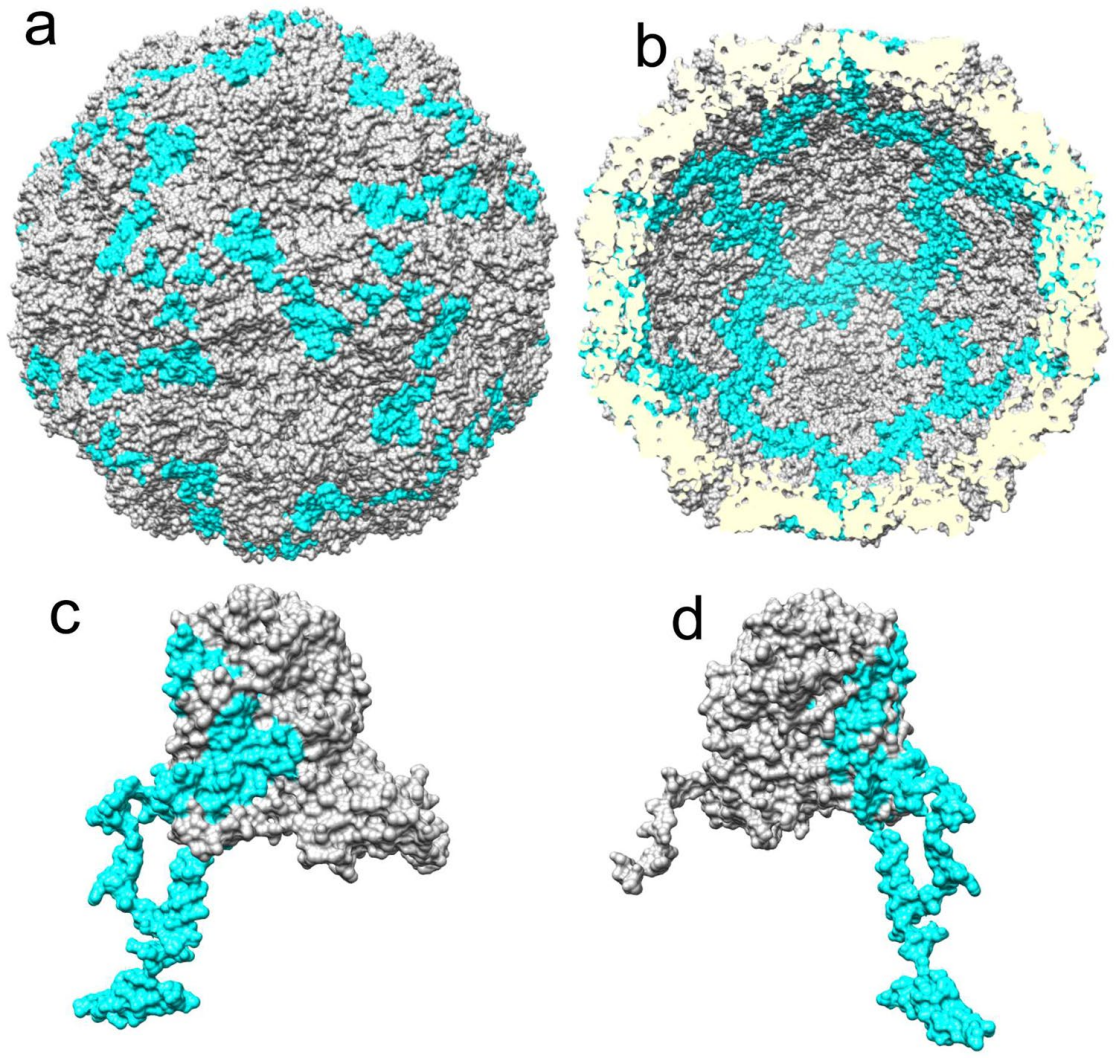
RCAP of capsid protein VP0. RCAP results of recombinantly-expressed VP0 are shown on the HPeV1 model (PDB: 4z92). (**a**) Peptides derived from VP0 identified to contact RNA that localize to the outer capsid surface whereas (**b**) shows the peptides on the inner capsid surface. (**c**) Location of peptides from VP0 that contact RNA on a monomer of VP0 with a view corresponding to the outer capsid surface whereas (**d**) A view of VP0 that corresponds to the inner capsid surface.

The purified VP0 was subjected to RCAP in the presence of sub-genomic HPeV1 RNA (Table 3). Unlike for VP1, a sharp increase in the number of peptides recovered was observed for recombinant VP0 when compared to whole virion experiments. Coverage increased from 49% to 67–78%, and the number of missed cleavages increased from 4 to 6 for the shortest sub-genomic RNA. The unique VP0 residues found to interact with RNA exclusively in experiments with recombinant VP0 were 175–199, 205–226 and 235–278. The atomic model of the virion reveals that residues 205–226 and 262–278 are buried in the pentamer at the interfaces between proteins, and 175–199 and 235–261 are on the outer surface of the capsid (Fig. 3). The highly reproducible identification of these residues, and the accompanying missed cleavages in RCAP experiments with the recombinant VP0 but not with whole virions, and their inaccessible location in the atomic model of the virion, suggest an RNA-binding role for these regions before final assembly of the capsid. The N-terminal disordered regions are recovered in all recombinant VP0 RCAP experiments, in contrast to the same experiments with recombinant VP1. A single, short 10-residue peptide at the C terminus is the only peptide not recovered in recombinant VP0 RCAP which was present in the whole virus RCAP (Tables 1 and 3). As with VP1 protein RCAP, varying the sub-genomic RNA length had a negligible effect on the regions identified in protein RCAP.

**Table 3:** Summary of peptides derived from capsid protein VP0 that were crosslinked to sub-genomic HPeV1 RNA.

Sub-genomic sequence length (n.t.)	Amino acid sequence^a^
1576	METIKS**IADM ATGVVSSVDS TINAVNEK**VE SVGNEIGGNL LTK**VADDASN ILGPNCFATT AEPENkNVVQ ATTTVNTTNL TQHPSAPTMP FSPDFSNVDN FHSMAY**DITT GDKNPSKLVR LETHEWTPSW AR**GYQITHVE LPkVFWDHQD** **kPAY**GQSRYF AAVRCGFHFQ VQVN**VNQGTA GSALVVYEPk PVVTYDSkL**E FGAF**TNLPHV LMNLAETTQA DLCIPY**VADT NYVK**TDSSDL GQLkVYVWTP LSIPTGSANQ VDVTILGSLL QLDFQNPR**VF AQDVNIYD*N*
2335	METIKSI**ADM ATGVVSSVDS TINAVNEkVE SVGNEIGGNL LTKVADDASN ILGPNCFATT AEPENKNVVQ ATTTVNTTNL TQHPSAPTMP FSPDFSNVDN FHSMAYDITT GDkNPSK**LVR LETHEWTPSW AR**GYQITHVE LPkVFWDHQD** **kPAYG**QSRYF AAVRCGFHFQ VQVN**VNQGTA GSALVVYEPk PVVTYDSkL**E FGAF**TNLPHV LMNLAETTQA DLCIPY**VADT NYVK**TDSSDL GQLKVYVWTP LSIPTGSANQ VDVTILGSLL QLDFQNPR**VF AQDVNIYD*N*
3037	METIK**SIADM ATGVVSSVDS TINAVNEK**VE SVGNEIGGNL LTK**VADDASN ILGPNCFATT AEPENkNVVQ ATTTVNTTNL TQHPSAPTMP FSPDFSNVDN FHSMAY**DITT GDKNPSKLVR LETHEWTPSW AR**GYQITHVE LPKVFWDHQD** **kPAYG**QSRYF AAVRCGFHFQ VQVNVNQGTA GSA**LVVYEPK PVVTYDSKL**E FGAF**TNLPHV LMNLAETTQA DLCIPY**VADT NYVK**TDSSDL GQLkVYVWTP LSIPTGSANQ VDVTILGSLL QLDFQNPR**VF AQDVNIYD*N*

^a^The RCAP peptides identified are in bold. The missed cleavages are shown lowercase and highlighted in bold-underline. The disordered regions not identified in the atomic model are underlined.

**Figure 4:**
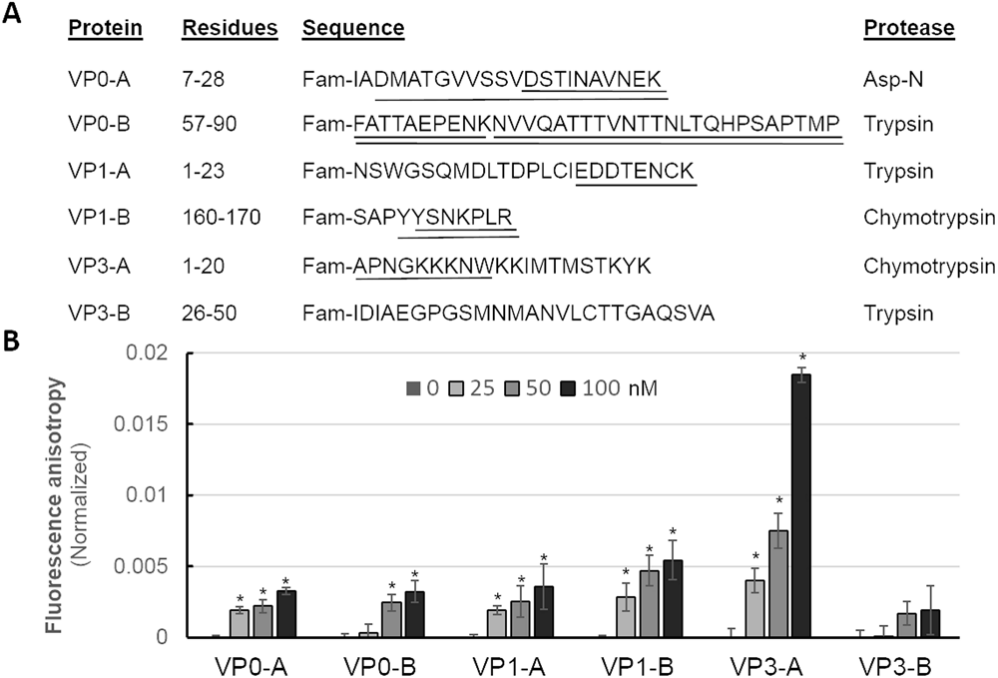
Intrinsically disordered regions in VP0, VP1 and VP3 can interact with HPeV1 RNA. (**A**) RCAP results of peptides from VP0, VP1, and VP3 that could interact with the HPeV1 RNA. The residues in the peptides crosslinked to RNA are underlined. The VP0-A, VP1-A and VP3-A peptides were all missing from the X-ray structure and predicted to be intrinsically disordered. The VP0-B, VP1-B and VP3-B peptides were all modelled in the X-ray structure. (**B**) Peptides from intrinsically disordered regions of VP0, VP1, and VP3 can bind to HPeV1 RNA in a fluorescence anisotropy assay. Each error bar shows one standard deviation of three independent measurements. The asterisk denotes the samples whose anisotropy values differ from the anisotropy value of the peptide alone by a p value of <0.02 in the Student’s T-test.

Whole-virion RCAP suggested that the N-terminal regions of VP0, VP1 and VP3 contact the encapsidated RNA. All of these regions were not resolved in the HPeV1 atomic model, and were predicted to be intrinsically disordered (Supplementary Figure 3). To further confirm that the intrinsically-disordered sequences identified in VP0, VP1 and VP3 can interact with RNA, and to more narrowly define the binding residues, peptides were synthesized corresponding to the disordered N-terminal regions (A-suffixed peptides in Fig. 4) and to structured regions elsewhere in the capsid proteins (B-suffixed peptides in Fig. 4). All peptides overlapped regions that were found to bind viral RNA in whole-virion RCAP experiments. Regions represented by VP0-A and VP0-B were found to bind sub-genomic viral RNA in protein RCAP experiments, while regions in VP1-A and VP1-B did not. The peptides were synthesized with a fluorescence tag and their RNA binding properties were examined using both the RCAP assay (Fig. 4A, Supplementary Figure 4A) and fluorescence anisotropy (Fig. 4B). Use of proteases that cleaved at other residues allowed us to further delineate regions within these sequences competent for RNA binding. Peptides containing cysteine residues were alkylated immediately prior to digestion using bromoethylamine, which converts cysteine residues to aminoethylcysteine, which are susceptible to proteolysis by trypsin^17,18^. RCAP analysis of these peptides verified that each of the N-terminal disordered regions bound viral RNA, as did two peptides derived from structured regions in VP0 and VP1. The peptide-RCAP experiment further defined the RNA-interacting residues of these sequences. Both of the peptides derived from VP0 were robust RNA binders with virtually the entire peptide sequence recovered in RCAP. The same was true of the structured peptide derived from VP1. In contrast, the peptide-RCAP experiment with the N-terminal disordered region of VP1 only recovered the C-terminal half of the peptide corresponding to residues 15–23, and the experiment with disordered VP3 sequence recovered only the N-terminal half of the peptide corresponding to residues 1–9. (Fig. 4A, Supplementary Figure 4B). The VP3-B peptide was not recovered (Fig. 4A). These results demonstrate that the intrinsically disordered regions of VP0, VP1 and VP3 do not require an assembled capsid or to be part of a structured protein to contact the sub-genomic HPeV1 RNAs.

Fluorescence anisotropy was used to examine the binding of these peptides to viral RNA (Fig. 4B). Binding with the sub-genomic RNAs resulted in an increase in anisotropy which was observed (p < 0.02) at all RNA concentrations for VP0-A, VP1-A, VP1-B, and VP3-A as well as at 50–100 nM RNA for VP0-B (Fig. 4B). The 3–4 fold increase in anisotropy observed in the VP3-A peptide can be explained by the presence of predicted RNA-binding residues 17–19 in this peptide. No significant increase in the anisotropy was observed for the peptide VP3-B, despite containing predicted RNA-binding residues 41 and 44. These results further confirm that intrinsically disordered regions of VP0, VP1, and VP3 can bind HPeV1 RNA and further bolster confidence in the RCAP data.

The three sets of RCAP experiments allowed us to mimic putative stages of virion capsid assembly and explore the importance of primary, tertiary and quaternary protein structure in binding RNA. Results from the protein RCAP experiments involve only isolated capsid proteins and viral RNA, and as such represent the earliest steps in assembly, before pentamer formation. Whole-virion RCAP represents the final form of the infectious virus, and so RNA-contacting residues observed here have the advantage of being stabilized by the overall quaternary structure of the assembled capsid. RNA-contacting sites gained or lost along the way to capsid assembly might give some clue as to the underlying assembly mechanism. One such residue identified in this work is 202 of VP1. This residue was found to be in contact with encapsidated viral RNA in the crystal structure of the virus^3^, and as well was found to be critical for virus viability in a tissue culture infectivity assay^4^. Similarly, this residue was identified in whole-virion RCAP, but was notably missing in analysis with recombinant protein. Elsewhere in VP1, the N-terminal disordered regions were found to bind RNA in whole-virion RCAP and in peptide-RCAP, but were similarly missing from the protein RCAP data. These findings suggest that VP1-RNA contacts are common in the assembled capsid, but are rather limited early in assembly.

In contrast, VP0-RNA contacting residues appear to have a role in early stages of capsid assembly. This was especially striking for the C-terminal third of the protein (residues 175–278), which was recovered consistently and with associated missed cleavages in protein-RCAP experiments, but was missing in whole-virion RCAP. In the atomic model of HPeV1, this region is unavailable to the encapsidated RNA. It stands to reason that this region could have a role early in assembly, but these contacts are lost later on, presumably after the pentamer has been formed and VP1 and VP3 are available to help stabilize pentamer-RNA binding. The N-terminal disordered region of VP0 is present in both whole-virion and protein-RCAP, again in contrast to VP1 where it only appears in whole-virion RCAP. A role for the N- and C-terminal regions of VP0 early in capsid assembly is attractive because VP0 is the first sequence synthesized during polyprotein translation, and would have the earliest and least-encumbered access to the viral mRNA. Such a transient interaction might help in the initial nucleation of capsid assembly. Subsequently, the VP3 N-terminus interacts with four other VP3 N-termini to form the VP3 annulus stabilizing the pentamer (Supplementary Movie 1). Formation of the pentamer creates new RNA binding sites formed from VP1 and VP3 which have high affinity for the multiple PS present on the genome^4^. At this stage collapse of the genome will bring the pentamers spatially close together on the genome^19^, allowing growth of the capsid that requires free VP0 N-termini for the inter-pentameric interactions observed in the virion structure.

Although technical challenges prevented us from performing a protein RCAP experiment on isolated VP3, the analysis of peptides derived from VP3 sequences in peptide RCAP and RNA fluorescence anisotropy provides some details. Residue 44 of VP3, like residue 202 of VP1, was predicted to bind viral RNA in the viral atomic model and confirmed in an *in vitro* infectivity assay. This residue was recovered in whole-virion RCAP, but was not recovered in peptide RCAP nor found to significantly increase the anisotropy of RNA fluorescence. Similarly, the predicted and confirmed RNA-binding residue 19 was in close proximity to recovered peptides in whole-virion RCAP. However, this residue was not recovered in peptide RCAP, which instead indicated that the N-terminal 10 residues had the highest affinity in the primary sequence for binding RNA. Generally, the previously-observed RNA-binding residues of the HPeV1 capsid appear to only contact RNA in RCAP experiments in the quaternary structure of the assembled capsid. Hence the high affinity binding pocket in the capsid for the packaging signal cannot be the nucleation point for virus assembly if nucleation happens prior to pentamer formation. A novel finding in this work is that the N-terminal disordered regions of VP0 and VP3 can contact viral RNA in the absence of an assembled capsid, and are thus potential early events in the capsid-RNA co-assembly route. The highly differential RNA-binding properties of VP0 in particular suggest that several protein-RNA contacts are gained and lost during assembly.

Many of the steps proposed here in our assembly model need to be tested. The concept of controlled assembly stages, regulated through multiple protein and nucleic acid interactions has been illustrated in other more tractable virus systems. In the positive-sense, single-stranded RNA virus, Alfalfa mosaic virus the N-terminus of the capsid protein and RNA co-fold upon interaction with each other priming the viral RNA for replication^20^. Similarly, Brome mosaic virus has an intrinsically disordered N-terminal tail that interacts differentially with encapsidated RNA, and can recognize specific RNA motifs in the BMV genomic RNA^21,22^. Both MS2 and Turnip crinkle virus form capsid protein dimers where the dimer conformation is altered by RNA binding, and is essential for capsid assembly to occur, limiting the number of potential assembly pathways^23–25^.

In other picornaviruses, an ordering of the encapsidated RNA likely occurs prior to the proteolytic processing of VP0 to generate VP2 and VP4. After the VP0 cleavage, the resulting VP4 and the N-terminus of VP2 are rearranged within the capsid, hence losing their ordered interactions with the RNA. Far fewer bases are ordered and are in base-stacking interactions with VP2 in icosahedrally-symmetric X-ray structures of a poliovirus and a rhinovirus^26,27^. RCAP analysis could be useful in exploring the dynamic RNA-protein interactions during assembly in the picornaviruses that undergo cleavage of VP0 to VP2 and VP4.

The results in this work demonstrate that intrinsically disordered sequences in the HPeV1 capsid play important roles in contacting the encapsidated RNAs. Intrinsically disordered sequences are highly abundant in viral capsid proteins^28^. In enveloped positive-sense RNA viruses such as flaviviruses and retroviruses, interactions with viral RNA are positively correlated with virulence^29,30^. In non-enveloped positive-sense RNA viruses like brome mosaic virus, the intrinsically disordered capsid protein N-terminus is known to associate specifically with viral RNA so that only the viral RNA are encapsidated during assembly^31^.

Additionally, the work establishes a role for VP0 in binding the viral RNA which was not observed in the X-ray structure of HPeV1, and provides some clues as to the sequence of protein-RNA binding events that eventually lead to capsid assembly. A general scheme of an early role for VP0 and a later or capsid-stabilized role for VP1 in binding the viral genome seems evident.

In conclusion, although the identification of VP0-RNA interaction via VP0 disordered N-terminus by RCAP is thus far seen only in parechoviruses, probably *en route* to assembly, we hypothesize that similar interactions will be important in the assembly of many other picornaviruses too.

## Methods

### Virus growth and purification

HPeV1 Harris strain was cultured in HT29 cells (SIGMA) and purified by cesium chloride gradient as described previously^2,4^. The virus was buffer exchanged using 100 kDa membrane filters (Amicon) to 20 mM Hepes, pH 8.0, 150 mM NaCl and 2 mM MgCl_2_.

### Capsid proteins expression and purification

VP0 was cloned in pCOLD-I (Takara Bio) which has an N-terminal His-tag. The primers used for cloning were:

5′-gaagaactcgagATGGAGACAATTAAGAGTATTG-3′; and

5′-gccgccaagcttTCAATTATCATATATGTTGAC-3′

The restriction sites for enzymes XhoI and HindIII are underlined in the primer sequence, respectively, and gene-specific regions in uppercase. The PCR running conditions were 98 °C (3 min), 80 °C (1 min), 55 °C (10 s), 72 °C (1 min), [98 °C (10 s) + 55 °C (10 s) + 72 °C (35 s)] × 29, 72 °C (10 min), 4 °C. The expression was performed in BL21 (DE3) by induction with 1 mM IPTG at 15 °C for 18 h. The cell pellet was resuspended in 20 mM bicine, pH 8.0, 150 mM NaCl, 2 mM dithiothreitol, 1 EDTA-free protease inhibitor tablet (ThermoFisher Scientific), 0.1 mg/mL lysozyme, ~25 units of benzonase (Merck Millipore) and kept on ice for 30 min. The cells were lysed at 15000 psi for 6 min in an Emulsiflex C3 (Avestin) high pressure homogenizer. The supernatant was added to nickel-bound agarose beads and VP0 was eluted from the beads with 1 M imidazole in 20 mM bicine pH 8.0 and 150 ml NaCl. The eluted VP0 was further purified by gel filtration on a superdex 75 10/300 GL column (GE Healthcare Life Sciences) in 20 mM bicine pH 8.0 with 150 mM NaCl.

The VP1 pET102 plasmid (ThermoFisher Scientific) was a kind gift from Katja C. Wolther, Amsterdam Medical Centre^32^. This plasmid has a His-patch thioredoxin on its N-terminus and 6 histidines on its C-terminus. The plasmid was transformed in *E coli* BL21 (DE3) for expression and induced with 1 mM IPTG at 18 °C for 18 h. The cells were lysed in 20 mM Tris-HCl pH 7.5, 150 mM NaCl, 1 EDTA-free protease inhibitor tablet (ThermoFisher Scientific), 0.1 mg/mL lysozyme, ~25 units of benzonase (Merck Millipore) by keeping on ice for 30 min followed by sonication for 10 min in pulse mode. The lysed cells were centrifuged and the pellet was washed twice in 20 mM Tris-HCl pH 7.5, 0.5 M NaCl, 5 mM imidazole, 0.5% Triton X-100 and resuspended in solubilization buffer: 20 mM Tris-HCl pH 8.0, 0.5 M NaCl, 8 M urea. The resuspended pellet was sonicated for 10 min in pulse mode. The soluble protein was recovered by centrifugation in rotor A-4-62 (Eppendorf 5810 R centrifuge) at 4000 rpm, 30 min and 4 °C. The chromatography and on-column folding was performed on a 1 mL HisTrap column (GE Healthcare Life Sciences). The solubilized protein was loaded onto the solubilization-buffer equilibrated column using a P1 pump. The column was washed with 5 column volumes of 20 mM Tris-HCl pH 8.0, 0.5 M NaCl, 8 M urea, 20 mM imidazole and returned to solubilization buffer. The column was then installed onto an Äkta FPLC system and a 20 ml linear gradient was run from 8 M urea (20 mM Tris-HCl pH 8.0, 0.5 M NaCl) to 0 M urea (20 mM Tris-HCl pH 8.0, 0.5 M NaCl). A step gradient of 5 mM, 10 mM, 20 mM, 40 mM, 60 mM, 300 mM and 500 mM imidazole in 20 mM Bicine pH 8.0, 500 mM NaCl was used to elute the on-column folded VP1 protein. Protein purity was assessed by SDS-polyacrylamide gel electrophoresis (Supplementary Figure 5).

The refolding of VP0 and VP1 was confirmed by Attenuated Total Reflectance Fourier Transform Infrared (ATR-FTIR) spectroscopy (Smiths Detection DuraSamplIR II and Bruker IFS 66/S). The FPLC fractions containing protein eluting at 1 or 0.5 M imidazole (VP0 and VP1, respectively) were assayed by pipetting 2 µL onto the ATR crystal surface and drying with forced air. Fractions that did not contain protein served as blanks. To obtain a reference spectrum of denatured peptide, aliquots of VP1 fractions and non-protein containing fractions were mixed 1:1 with 100% trichloroacetic acid (TCA) and assayed as wet drops to obtain spectra of denatured protein.

### *In vitro* transcription

The HPeV1 sub-genomic RNAs used in RCAP and RNA fluorescence anisotropy experiments were PCR amplified from an HPeV1 infectious cDNA clone^33^ with T7 promoter region present in the forward primer. Sub-genomic lengths of 1576, 2335 and 3037 nucleotides correspond to the 5′UTR-VP0, 5′UTR-VP3 and 5′UTR-VP1 regions of the HPeV1 genome. The forward primer used for amplifying all the three sub-genomic regions is:

5′-taatacgactcactatagggTTTGAAAGGGGTCTCCTAGAGAG-3′.

The reverse primers are:

5′-gccgccaagcttTCAATTATCATATATGTTGAC-3′ (for 1576 n.t.);

5′-gccgccaagcttTCAATATGGACTCTGATTTG-3′ (for 2335 n.t.);

5′-gaagccaagcttTCACTGGAATGTAACAACAG-3′ (for 3037 n.t.).

The gene-specific regions are in uppercase. The PCR was performed for 25 cycles with a denaturation temperature of 98 °C, annealing temperature of 55 °C and extension temperature of 72 °C. The PCR products were used for *in vitro* transcription of RNA using MEGAscript T7 transcription kit (ThermoFisher Scientific, Supplementary Figure 5A). The transcription was done at 37 °C, for 2 h. The RNA purification was done using the MEGAclear transcription clean-up kit (ThermoFisher Scientific) and RNA quality checked by agarose gel electrophoresis (Supplementary Figure 5).

### RCAP

The RCAP assay was done as described previously^11^, using two moles of recombinant protein to one mole of RNA. Formaldehyde was then added to a final concentration of 0.1% and incubated for 10 min at room temperature. Glycine (0.2 M) was added to quench the formaldehyde and incubated for 10 min at room temperature, then crosslinked protein-RNA complexes were subjected to trypsin digestion using sequencing-grade trypsin (Trypsin Gold, Promega) for 16 hours using a 1:20 w/w ratio of enzyme to capsid. RNA-peptide complexes were then selectively precipitated using lithium chloride, and to facilitate mass spectrometric analysis peptide-RNA conjugates were reversed by supplementing the buffer with 150 mM sodium chloride and heating at 65 ^ο^C for 1 h. Control reactions lacking formaldehyde, and when using the recombinant protein lacking RNA, were performed in parallel.

RCAP assays performed with chemically synthesized peptides with an N-terminal carboxyfluorescein (Zhejiang, China). Each peptide was purified using HPLC to at least 95% purity and the molecular mass was confirmed by mass spectrometry to be accurate to within 1 Dalton. The peptides were present at two molar excess to the HPeV1 RNA and crosslinked as described above. To facilitate ionization and proteolysis, cysteine-containing peptides were supplemented with 200 mM Tris-HCl (pH 9.0), 5 mM TCEP, and alkylated with 20 mM bromoethylamine prior to digestion as described previously^17,18^. The crosslinked peptide-RNAs were digested with either 1 μg trypsin, chymotrypsin, or Asp-N as specified (Pierce MS-grade protease) overnight at 37 °C. Controls included RNAs and peptides without formaldehyde, and proteolyzed samples.

For the virion RCAP data, peptides were analyzed using an LTQ Velos Pro dual-pressure linear ion trap mass spectrometer (ThermoFisher Scientific). Tryptic digests were injected into a C18 column (Dionex UltiMate 3000 HPLC) and eluted using a linear gradient of 2–45% acetonitrile in water with 0.1% formic acid, over a 90 minute gradient. The flow rate was 50 uL/min, and effluent was electro-sprayed into the LTQ. Tandem mass spectra were obtained using collision-induced dissociation. Experiments using recombinant protein were analyzed on an LTQ Orbitrap mass spectrometer (ThermoFisher scientific), and separated similarly using an Accela HPLC. Peptide RCAP data was generated on a Bruker Autoflex III MALDI-ToF and analyzed using GPMAW (General Protein/Mass Analysis for Windows, Supplementary Figure 4).

Database searches used searchGUI and the peptide sequences were compiled with peptideshaker^34,35^. Databases were created using HPeV1 protein sequences appended to a FASTA file containing the uniprot human proteome and the common repository of adventitious proteins database^36^. Linear ion trap data was searched using a 0.3 Da mass error, and Orbitrap using 10 ppm error. Peptides appearing in the negative controls were used to gauge the quality of LiCl precipitation, and results were discounted if peptides were detectable. Only identifications with a confidence score of 98% or better were included. The identified peptides for the virion RCAP are listed in Supplementary Table 1 and the top 12 hits for each protein were considered as most significant. This accounted for 81% of the unique observations. Disorder predictions were retrieved from the PONDR server (http://www.pondr.com/) using the VL-XT algorithm^37^.

### Fluorescence Anisotropy Assay

Peptide binding to HPeV1 RNA used peptides containing an N-terminal carboxyfluorescein. HPeV1 RNA was titrated into 100 µL solution of peptide (0.1 µM) to final concentrations 25–100 nM and the results were normalized to the anisotropy of the peptide alone. Fluorescence anisotropy data was generated on a Synergy H1 microplate reader (BioTek) and calculations were performed in Gene 5 software (Biotek). Pairwise T-Tests were performed using Microsoft Excel.

### Data availability

The datasets generated and analyzed during the current study are available from the corresponding authors on request.

## Electronic supplementary material

Supplementary information

Supplementary Movie 1
