# Supplementary material for: Intrinsically-disordered N-termini in human parechovirus 1 capsid proteins bind encapsidated RNA
Source: Sci Rep. 2018 Apr 11;8:5820. doi: 10.1038/s41598-018-23552-7 (PMC5895611; doi:10.1038/s41598-018-23552-7)
Supplement: Supplementary file 1 — Supplementary information [file 41598_2018_23552_MOESM1_ESM.docx]

# Supplementary information

**Intrinsically-disordered N-termini in human parechovirus 1 capsid proteins bind encapsidated RNA**

Shabih Shakeel^1,3^, Mark Hazelbaker^2^, James D. Evans^1^, C. Cheng Kao^2^, Robert C. Vaughan^2*^ and Sarah J. Butcher^1*^

^1^Institute of Biotechnology and Research Programme in Molecular and Integrative Biosciences, Faculty of Biological and Environmental Sciences, University of Helsinki, 00790 Helsinki, Finland

^2^Department of Molecular and Cellular Biochemistry, Indiana University, Bloomington, IN 47405, USA

^3^Current address: MRC Laboratory of Molecular Biology, Francis Crick Ave, Cambridge CB2 OQH, UK

^*^Corresponding authors

Emails: sarah.butcher@helsinki.fi (SJB), robvaugh@indiana.edu (RCV)

Supplementary Table 1. List of all peptides confidently identified in virion RCAP, with the top twelve hits highlighted in yellow for each protein.

#
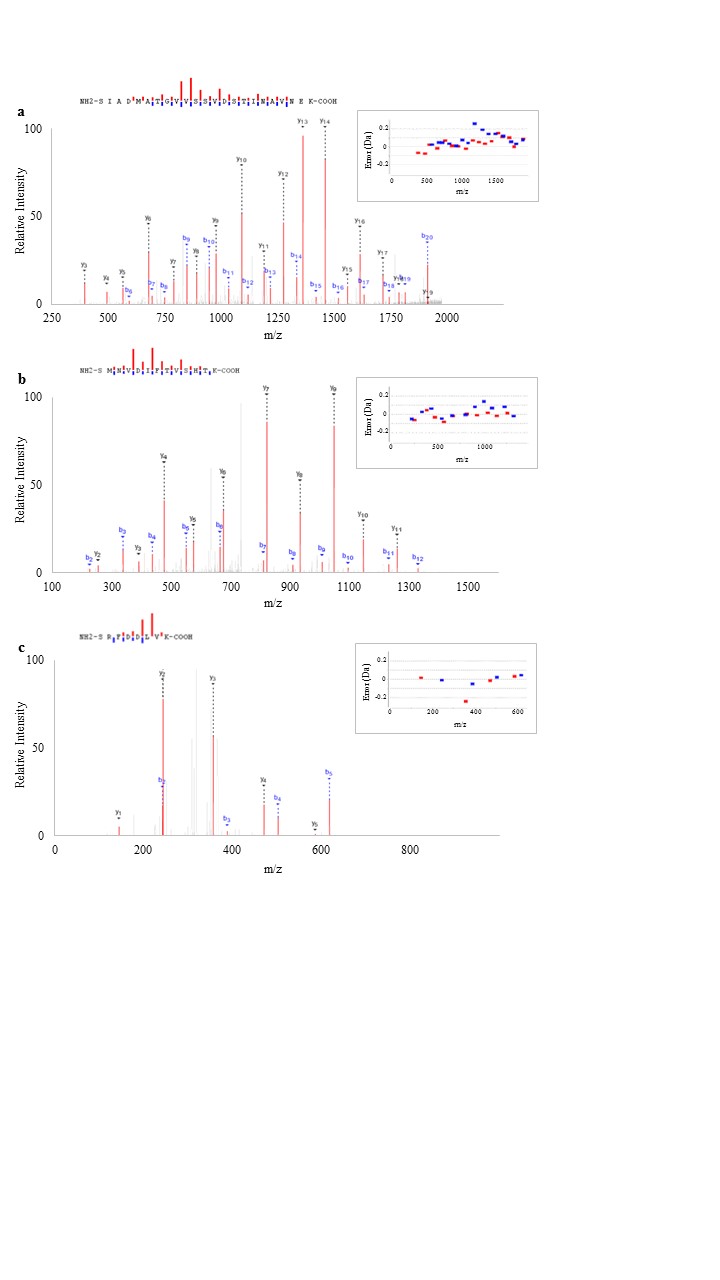


# Supplementary Figure 1. Peptides recovered by whole-virion RCAP

Representative MS/MS spectra from the HPeV1 virion RCAP assay. Peptide assignments derived from VP0 (panel A), VP1 (panel B), and VP3 (panel C) are depicted schematically above each spectra. Red bars indicate y ions and blue b ions. The size of each bar is relative to the intensity of the match in each spectra. Images were exported directly from PeptideShaker, and insets represent ms/ms mass error (Da).


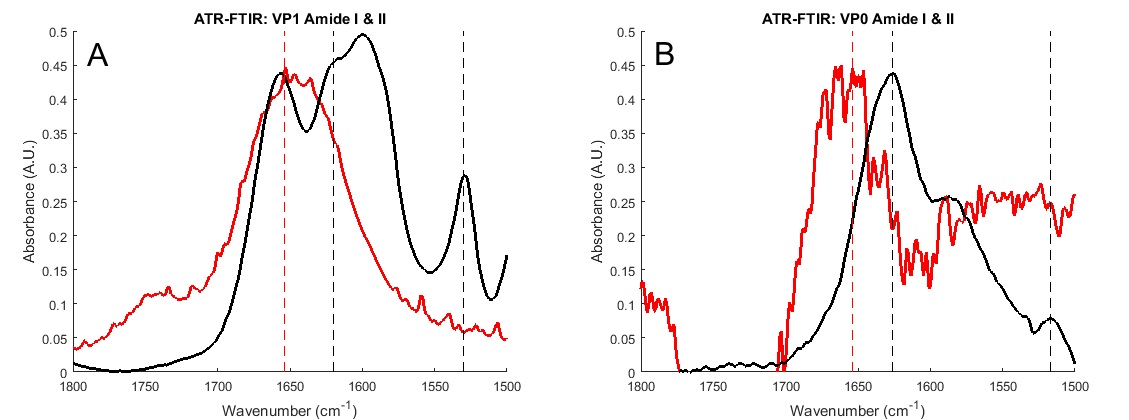


# Supplementary Figure 2. ATR-FTIR spectra of purified capsid proteins VP1 and VP0.

The amide I and amide II regions of the infrared spectra of A.) purified VP1 *(black trace)* and TCA-denatured VP1 *(red trace)*; and B.) purified VP0 *(black trace*) and TCA-denatured VP0 *(red trace*). Dashed vertical lines denote peak positions corresponding to secondary-structure dependent amide I absorption: red lines at 1654 cm^-1^ for unstructured peptide, black at 1620 cm^-1^ (VP1) or 1626 cm^-1^ (VP0) for absorption due to β-sheet amides. Amide II peaks are likewise denoted with vertical dashed black lines at 1530 cm^-1^ (VP1) or 1517 cm^-1^ (VP0) and are absent in the TCA-denatured samples.


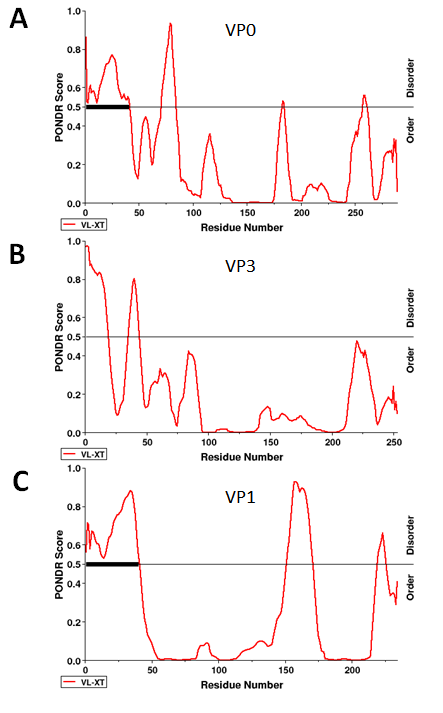


# Supplementary Figure 3. PONDR analysis of viral capsid proteins

A.) VP0, B.) VP3, C.) VP1. A score of more than 0.5 predicts disorder. The regions in VP0 and in VP1 which have over 39 residues in a row predicted to be disordered, are marked with a thick black bar.


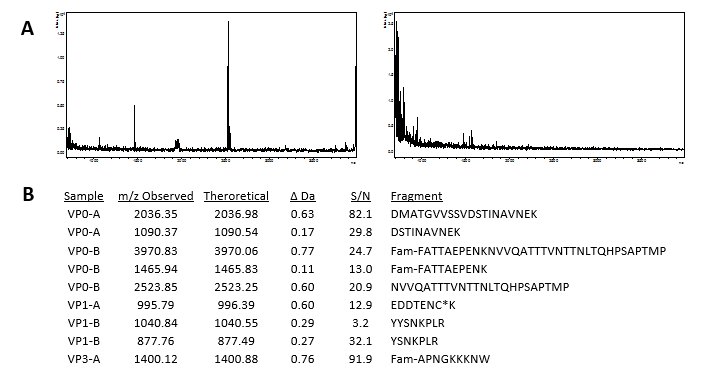


# Supplementary Figure 4. Peptide RCAP Mass Assignments.

A.) Representative RCAP MALDI spectra from the peptide RCAP experiment performed with the VP0-B peptide *(left)*, and the control reaction lacking formaldehyde *(right)*. B.) Theoretical digests of all synthesized peptides were performed using GPMAW (Lighthouse Data, Hanstholm, Denmark) and matched against observed masses.

#
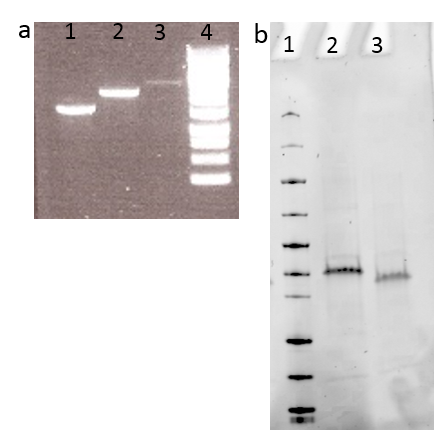


# Supplementary Figure 5. RNA and protein gels.

A.) 1% agarose gel stained with SybrSafe DNA gel stain (Invitrogen S33102). Lane 1: HPeV1 subgenomic transcript of 1576 nucleotides; 2: 2335 n.t.; 3: 3037 n.t., and; 4: GeneRuler 1 kb DNA ladder (ThermoScientific SM0312). B.) 4-20% TGX stain-free SDS gel (BioRad; Cat#456-8093) UV exposed for 5 min before imaging. Lane 1: PageRuler broad range unstained protein ladder (ThermoScientific 26630); 2: purified VP0; 3: purified VP1.

# Supplementary Movie 1. The inner capsid-surface peptides identified by RCAP.

The movie shows the HPeV1 pentamer (PDB: 4Z92) in ribbon representation. The thicker sections of the ribbon represent the inner capsid-surface peptides recovered in whole virion RCAP analysis. The VP0 is colored cyan; VP1, magenta and; VP3, orange.
